# The Effects of a Digital Mental Health Intervention in Adults With Cardiovascular Disease Risk Factors: Analysis of Real-World User Data

**DOI:** 10.2196/32351

**Published:** 2021-11-19

**Authors:** Robert M Montgomery, Eliane M Boucher, Ryan D Honomichl, Tyler A Powell, Sharelle L Guyton, Samantha L Bernecker, Sarah Elizabeth Stoeckl, Acacia C Parks

**Affiliations:** 1 Happify Health New York, NY United States

**Keywords:** digital mental health, digital health, mobile apps, mobile health, internet-based intervention, happiness, subjective well-being, anxiety, cardiovascular health, high blood pressure, high cholesterol, diabetes, cardiovascular disease risk, real-world data

## Abstract

**Background:**

The American Heart Association has identified poor mental health as a key barrier to healthy behavior change for those with cardiovascular disease (CVD) risk factors such as high blood pressure, high cholesterol, and diabetes. Digital mental health interventions, like those delivered via the internet to computers or smartphones, may provide a scalable solution to improving the mental and physical health of this population. Happify is one such intervention and has demonstrated evidence of efficacy for improving aspects of mental health in both the general population and in users with chronic conditions.

**Objective:**

The objectives of this analysis of real-world data from Happify users with self-reported CVD risk factors, including high blood pressure and cholesterol, diabetes, and heart disease, were to examine whether these users would report improvements in subjective well-being and anxiety over time (H1) and use of Happify as recommended would be associated with significantly greater improvement in subjective well-being and anxiety over time compared to less-than-recommended usage (H2).

**Methods:**

Data were obtained from existing Happify users who reported the aforementioned CVD risk factors. The sample included 1803 users receiving at least 6 weeks’ exposure to Happify (ranging from 42 days to 182 days) who completed at least one activity and two assessments within the app during that time. Subjective well-being was assessed with the Happify Scale, a 9-item measure of positive emotionality and life satisfaction, and anxiety was assessed with the Generalized Anxiety Disorder 2 (GAD-2). To evaluate H1, changes over time in both outcomes were assessed using mixed effects linear regression models, controlling for demographics and usage. For H2, an interaction term was added to the models to assess whether usage as recommended was associated with greater improvement over time.

**Results:**

Both hypotheses were supported. For both the Happify scale and GAD-2, the initial multivariable model without an interaction demonstrated an effect for time from baseline, and the addition of the interaction term between time and recommended use was significant as well.

**Conclusions:**

This analysis of real-world data provides preliminary evidence that Happify users with self-reported CVD risk factors including high blood pressure or cholesterol, diabetes, and heart disease experienced improved well-being and anxiety over time and that those who used Happify as recommended experienced greater improvements in these aspects of mental health than those who completed fewer activities. These findings extend previous research, which demonstrated that engagement with Happify as recommended was associated with improved well-being among physically healthy users and in those with chronic conditions, to a new population for whom mental health is especially critical: those at risk of developing CVD.

## Introduction

### Background

Cardiovascular diseases (CVDs) are the leading cause of death and disability worldwide, causing nearly one-third of all global deaths in 2019 [1] and trillions of dollars in projected global annual health care costs [2,3]. All told, in the United States, where health care expenditures are the greatest in the world, CVD is responsible for 17% of national health care expenditure [2,4]. Hypertension, or high blood pressure, as well as dyslipidemia (high cholesterol) and uncontrolled blood sugar (prediabetes or diabetes), are 3 of the most prevalent preventable risk factors for CVD, affecting 31%, over 50%, and 16.7% of adults worldwide, respectively [5-7]. Combined, high blood pressure, high cholesterol, and diabetes are responsible for the vast majority of cardiovascular-related deaths [8]. The Framingham Risk Function calculator, which models the contribution of various risks to the incidence of CVD, relies primarily on these 3 factors, in addition to age, gender, and smoking status, for producing risk estimates [9]. Economically, high blood pressure alone accounts for approximately 10% of global health care spending [10], with per capita annual health care costs estimated between US $4871 and US $11,238 [11]. Diabetes is estimated to cost US $827 million annually for global disease care [12], and the global burden of high cholesterol is estimated to be nearly US $4 billion annually [13]. Consequently, mitigating risk factors like high blood pressure, high cholesterol, and diabetes is critical to reducing the overall burden associated with CVD [14].

Self-management of physical health and psychological well-being is critical for reducing CVD risk and improving cardiovascular health [15-18]. The current first-line recommendation for managing CVD risk is adopting healthy lifestyle behaviors, including eating a healthy diet, not smoking, being physically active, and managing weight [19]. Additionally, consistent medication management, access to professional care, social support, and psychological health have been identified as important factors for success [1,16,17,20-23]. However, there are many barriers that can interfere with self-management [24,25]. In particular, poor mental health has been found to compromise many self-management behaviors, including medication adherence, adherence to cardiac rehabilitation programs, exercise, and healthy diet [26-30].

### Mental Health and Cardiovascular Health

The American Heart Association has identified poor mental health in particular as a key barrier to health behavior change [17]. Mental health has been defined as both the absence of psychological distress, including symptoms of anxiety, depression, stress, and associated mental disorders, and the presence of positive psychological well-being, which includes factors like life satisfaction, optimism, and positive emotion [31-33]. Research has demonstrated that psychological distress and positive psychological well-being are related, but distinct constructs that uniquely contribute to the prediction of various health outcomes [34,35], including cardiovascular health [36,37], and therefore a complete description of mental health and its relationship with CVD risk should include both.

There is a large body of research establishing a relationship between CVD and psychological distress [38]. Psychological distress is elevated for populations with poor cardiovascular health relative to those with better cardiovascular health [39], and mental disorders are more prevalent in those with CVD than in the general population [38]. Approximately 20%-30% of people with CVD or CVD risk factors may experience elevated symptoms of psychological distress, including depression or anxiety [40,41]. There is evidence that the relationship between psychological distress and cardiovascular health is bidirectional, such that elevated psychological distress negatively impacts cardiovascular health [42] and poor cardiovascular health increases the risk of psychological distress and even mental illness [43-46]. This may be explained, in part, by the finding that psychological distress impairs self-management and self-care activities, such as healthy eating and exercise, thereby undermining cardiovascular health [47]. Simultaneously, CVD risk factors may upregulate the intensity of the body’s inflammatory response system [48-51], which in turn, increases perceived stress and reduces psychological resilience [52].

There is also a well-established relationship between cardiovascular health and various aspects of positive psychological well-being, such as optimism, life satisfaction, and positive emotion [53]. Psychological well-being is lower for those with CVD risk factors than in those without such risk factors [54], and the frequent experience of positive emotion has been found to impact the successful prevention, management, and treatment of CVD [36,37]. In fact, positive psychological well-being has been found to protect against CVD risk, independent of common risk factors and psychological distress [53]. This may be because positive mood increases the frequency of key self-care and management activities in patients with chronic illnesses [55], including CVD [56]. It is clear that mental health, including both the absence of psychological distress and the presence of positive psychological well-being, is critically important to cardiovascular health.

Although there is ample research supporting the effectiveness of psychological interventions such as cognitive behavioral therapy (CBT) [57] for improving mental health [58,59], fewer studies have assessed whether mental health–focused interventions can positively affect cardiovascular health in patients at risk for CVD [60-63]. A meta-analysis including 35 randomized controlled trials (RCTs) examining psychological interventions in patients with chronic heart disease suggested that such interventions reduced the risk of cardiac mortality by an estimated 21% [64]. However, the authors noted that there was significant heterogeneity in quality and outcomes across studies and that there is still uncertainty regarding the magnitude of effects and the particular interventions or techniques that may benefit this population [64]. Another review found that, among face-to-face psychological interventions, CBT demonstrated the strongest evidence of positive impact on mental health and cardiovascular health in patients with CVD risk factors [62]. Mindfulness-based interventions and interventions promoting positive psychological well-being may also reduce CVD risk, though more research is needed to determine the details of when, why, and how they may do so [65-67]. There is some evidence to suggest that greater subjective well-being is associated with a number of positive physiological outcomes, such as increased longevity [67], though the strength and consistency of this association may vary across populations and contexts [68]. Even if the direct effect of most mental health interventions on CVD risk is small, the benefits of improved self-care behaviors and quality of life make these interventions indispensable to those with CVD risk factors [36].

### Barriers to Care and the Importance of Digital Interventions

Numerous barriers, including cost, stigma, and availability, limit people’s access to effective mental health interventions [69]. Digital interventions, like those delivered via the internet to computers or smartphones, can circumvent many of these barriers and may provide a scalable solution to supporting the physical and mental health of patients with CVD risks [70-74]. There are a number of digital health interventions that directly target self-management of CVD risks such as smartphone apps and wearable devices that focus on lifestyle behavior change, some of which have demonstrated efficacy in improving cardiovascular health outcomes [75-77]. Unfortunately, these interventions do not address the barriers introduced by mental health issues and may therefore be less effective for the increasing proportion of the population suffering from mental health difficulties each year [78-80]. As such, there is a need for scalable mental health interventions that are effective for this population. There is ample evidence indicating that digital mental health interventions such as internet-based CBT are safe and effective in general populations [81-83], but few of these interventions have demonstrated efficacy in improving aspects of mental health in patients with CVD risk factors [84-86]. One example is an internet-based CBT program that was adapted to patients with CVD and at least mild depression, which had a moderate to large effect on depression scores over 9 weeks (ds=0.62-0.86) compared with an online forum control group [86]. Other studies have evaluated the characteristics of digital mental health app users with CVD risk factors [77] and explored the feasibility and usability of digital interventions for mental and cardiovascular health [87]. However, most of these interventions lack evidence regarding their safety and effectiveness, and those that do have empirical support are generally not widely accessible [88,89]. Therefore, more research is needed on scalable, widely accessible digital mental health interventions in populations with CVD risk factors.

Happify is a mobile and web-based digital intervention designed to support mental and physical health through engagement with a variety of activities drawn from evidence-based treatments. Prior research has demonstrated Happify’s effectiveness in improving mental health and well-being. One RCT showed the positive mental health effects of Happify in a general population of US adults, finding that participants who completed a minimum 16 activities over 8 weeks improved their psychological resilience by 20.8% and reduced depression and anxiety symptoms by over 25%, effects twice as large as those observed in the active psychoeducation placebo control condition [90]. Additionally, a real-world naturalistic study of Happify in those with and without self-reported chronic conditions found that users with a chronic condition experienced significant improvement in subjective well-being over time (42-182 days from baseline) and that the trajectory of this change did not differ from those without a self-reported chronic condition [91]. Consistent with previous research, users who completed more activities, regardless of chronic condition status, had greater improvements in subjective well-being.

### Study Objectives

Although extant research suggests Happify users with chronic physical conditions experience significant improvements in subjective well-being over time [91], the previously published study combined all users with self-reported chronic conditions into a single group, and therefore, the specific impact on subpopulations, such as those with CVD risk factors, could not be observed. The previous study also only evaluated changes in positive aspects of mental health (subjective well-being) but not negative features (eg, anxiety) [91]. Therefore, the objective of this analysis of real-world user data was to expand on previous research by examining changes in both subjective well-being and anxiety over time in Happify users with self-reported CVD risk factors, including high blood pressure and cholesterol, diabetes, and heart disease. We hypothesized that (H1) Happify users with CVD risk factors will experience significant improvements in both subjective well-being and anxiety over time and (H2) users who engage with Happify at the recommended level (an average of 2 or more activities per week) will report significantly greater improvements in these mental health outcomes over time than those who completed fewer activities. While demonstrating improvement over time is a necessary first step, the second question is especially important as it would support stronger inferences about the relationship between engagement with Happify and improvements in mental health outcomes.

## Methods

### Recruitment

The sample consisted of users who found Happify via the Apple App Store or Google Play Store, internet search, digital advertisements, employee and health plan benefits programs, or other channels and signed up of their own accord. This study included data for 3 different subgroups of existing Happify users: consumer guests, who had free access to a limited version of the app; premium users, who paid for full access to the platform; and enterprise users, who received full access via their employer or health plan. The onboarding process for all new Happify users involves the following steps. After downloading the app or accessing the Happify website, all users complete an onboarding questionnaire, which contains questions about the user’s demographic characteristics as well as common intra- and interpersonal problems. One of these questions asks users to select all that apply from a list of conditions, including high blood pressure or cholesterol, heart disease, diabetes, migraine, psoriasis, rheumatoid arthritis, psoriatic arthritis, insomnia, eczema (atopic dermatitis), asthma, multiple sclerosis, cancer, arthritis, chronic pain, postpartum depression, or other. This item was used as a screening criterion for this study. Next, users receive an algorithmically generated recommendation for a “track” (a group of activities with a common theme) based on the challenges or conditions they reported during onboarding, though they are free to engage with any of the hundreds of available tracks in any order. After selecting a track, users can begin to complete activities.

Upon sign-up for Happify, users must agree to the terms of service and privacy policy, which includes the following statement: “Information that we collect about you also may be combined by us with other information available to us through third parties for research and measurement purposes, including measuring the effectiveness of content, advertising, or programs.” All data analyzed in this study were real-world data entered or generated by app users as part of the standard user experience and stored on secure company servers. Only anonymized data were extracted from the user database, and no personal data were submitted for scientific evaluation. Users were not offered any compensation to complete activities or assessments.

### Participants

Data were drawn from registered Happify users who reported having one or more of the following conditions: “High blood pressure or cholesterol,” “Diabetes,” or “Heart disease.” Data from users aged 18 years and older who created accounts between November 5, 2018 and May 31, 2021 (when data were queried) were considered for inclusion in the analysis (users were not asked about high blood pressure/cholesterol, diabetes, or heart disease prior to November 5, 2018). Users also had to meet the following inclusion criteria: complete at least 1 activity, complete no more than 3 activities before their baseline assessment, and complete at least 1 assessment in addition to baseline within 42-182 days (6 weeks to 6 months) from signup. This time window aligns with the time window used in our previous analysis of Happify users with self-reported chronic conditions [91], facilitating comparison. Naturalistic studies can yield notoriously messy data [92], and these criteria were selected in order to increase the interpretability of the results. Specifically, users who completed no activities were removed because any improvements they experienced could not be due to the use of Happify; users who completed more than 3 activities before the baseline were removed because their scores may not accurately represent their initial state before using Happify; and users without a follow-up assessment within the time window were removed because change over time cannot be assessed with only a single timepoint.

Between November 5, 2018 and May 31, 2021, there were 254,312 new sign-ups, among whom 18,905 reported at least one of the heart-related conditions. Of these, 4262 users completed at least 1 activity, at least 2 assessments, and no more than 3 activities before the first assessment. Restricting the sample to those with an assessment in the 42-182–day window reduced the sample to 2107. Finally, users who were missing multiple demographic variables due to a server error were removed, leaving a final sample of 1803.

### Intervention Description

A detailed description of the Happify platform, including screenshots, is available in previous research [90]. Briefly, Happify is a digital intervention designed to support mental and physical health through engagement with a variety of activities drawn from evidence-based treatments, including CBT [57], positive psychology [93], and mindfulness-based stress reduction [94]. These activities are generally brief, ranging from 2 minutes to ≥15 minutes, and are delivered in 5 media formats: (1) written activities, some of which are guided by a US-patented digital artificial intelligence coach (chatbot) called Anna; (2) audio recordings; (3) video recordings; (4) quizzes; and (5) cognitive training games. Some activities can be completed fully within the app (eg, psychoeducational quiz about happiness), while others require action outside of the app (eg, calling a friend or practicing a more adaptive response to an upsetting event or situation). Activities are grouped into 6 skills: savoring (eg, mindfulness-based activities), thanking (eg, gratitude-based activities), aspiring (eg, optimism and goal-setting activities), giving (eg, kindness and forgiveness activities), empathizing (eg, self-compassion and perspective-taking activities), and reviving (eg, physical activities). These activities are organized into 4-week tracks that address particular challenges or symptoms (eg, addressing negative thoughts or reducing stress). Each track contains approximately 30-40 activities subdivided into 4 parts. Users select a track of interest but can switch tracks anytime and can also access activities on-demand, separate from any track.

Previous research on Happify has indicated that completing at least 16 activities over 8 weeks, or an average of ≥2 activities per week, is associated with moderate increases in mental health outcomes, and thus, this is considered the minimum recommended level of use [90,95]; however, users are not directed or required to use Happify at a particular frequency. Two new activities are available each day, with the option to unlock a third new activity if at least one activity is completed that day, though users may complete as many instant play activities as desired.

### Outcome Measures

Happify includes regular assessments of different aspects of mental health, including anxiety and subjective well-being, and provides visual feedback to users via a graph that tracks their subjective well-being over time. One day after registering and every 2 weeks thereafter, all Happify users are invited to complete a well-being assessment called the Happify Scale and the Generalized Anxiety Disorder-2 (GAD-2) assessment [90,96,97]. Assessments are optional, and thus, there is considerable variability in the frequency and timing of assessment completion of these measures in the data analyzed for this study.

#### Subjective Well-Being

The Happify Scale has been described in detail in other publications [91,97]. Briefly, the Happify Scale is a 9-item measure with 2 subscales: a 4-item positive emotionality scale and a 5-item life satisfaction scale [97]. Items on the positive emotionality subscale are rated on a 5-point scale from “Never” to “Very often (almost every day),” and items on the life satisfaction subscale are rated on a 7-point scale from “Very dissatisfied” to “Very satisfied.” Scores are converted into percentages and thus range from 0 to 100, where higher scores on each subscale indicate greater positive emotionality and life satisfaction. Subscale scores are typically averaged together such that higher composite scores indicate greater subjective well-being. Scale validation using a general population sample from Amazon’s Mechanical Turk showed that scores between 46 and 49 corresponded to the 25th percentile, scores between 61 and 63 corresponded to the 50th percentile, and scores between 75 and 77 corresponded to the 75th percentile of the Happify Scale. Internal validation data indicated that composite scale scores had acceptable reliability (*α*=.89) and was strongly correlated with subjective happiness (*r*=0.78) [98] and anticorrelated with a measure of depressive symptoms (*r*=−0.7) [99].

#### Anxiety Symptoms

The GAD-2 is a 2-item initial screening tool for generalized anxiety disorder that consists of the first 2 questions of the GAD-7 [100]: “Over the last two weeks, how often have you been bothered by the following: (1) Feeling nervous, anxious, or on edge and (2) Not being able to stop or control worrying.” Responses are Likert-style from 0 to 3 (0=not at all*,* 1*=*several days*,* 2*=*more than half the days, and 3*=*nearly every day)*.* The scores for both items are summed for a total score range of 0-6. A total score of ≥3 is recommended as a cut-off for detecting generalized anxiety disorder in the general population, though clinical interviews are generally required for diagnostic purposes [101]. Though the GAD-2 is most often used as a screening tool, it has also been used to measure responsiveness to treatment effects in both clinical study and primary care settings [102]. Staples and colleagues [102] compared the short-form against the full version of several common mental health measurement tools, including the GAD-7, and found that the percentage change and within-person effect sizes were of similar magnitude across the 2 versions of the measure. Therefore, the GAD-2 may be a practical and effective means of measuring change in anxiety symptoms over time in the context of an intervention study.

### Statistical Analysis

To test both hypotheses, linear mixed effects (LME) models were fitted for each outcome, with time from baseline (in days) as a level 1 fixed effect predicting each assessment score. LME is capable of modeling change over time in longitudinal data that have a high degree of variability and heterogeneity [103]. As is typical with real-world data [103,104], our data were highly variable regarding the frequency and timing of assessments, and thus, LME was used to account for these factors. The Akaike information criterion was used to identify the best random effects model, and ultimately, all LME models were fitted with a random intercept and random slope for time. Because users had varying numbers of assessments at unequally spaced times, a continuous autoregressive error structure of order 1 was used.

To test the first hypothesis, that Happify users with CVD risk factors would experience significant improvements in mental health outcomes over time, models were fit to examine the fixed effect of time—that is, the coefficient representing whether and how quickly users decreased in anxiety and increased in subjective well-being—while controlling for person-level (ie, level 2) covariates. Person-level predictors included the total number of chronic conditions reported by the user at baseline (which could include heart-related conditions and other conditions), gender, age category, baseline GAD-2 or Happify Scale composite score (each predicting the other measure; eg, [91]), relationship status, whether the user had any minor-aged children, and a dichotomous variable that indicated whether a user completed the recommended use of an average of ≥2 activities per week. That is, a user was categorized as having reached the recommended use if the total number of activities completed between first and last assessment, divided by the number of weeks between assessments, was ≥2. This cutoff has been established in previous research as an approximate threshold for the minimum amount of engagement required to produce meaningful effects [90,91]. Coefficients for these person-level predictors represent whether they are associated with higher or lower mental health overall, but not whether they are associated with how quickly a person’s mental health improves.

To test the second hypothesis, that users who engage with Happify at the recommended use report significantly greater improvements in these mental health outcomes over time than those who use it less, an interaction between the user’s recommended use status and time was added to the model. The coefficient for this interaction represents how the change in mental health over time (ie, the time slope) differs between those who used at the recommended level and those who did not.

Assumptions of final models were evaluated via visual inspection of residual plots. For all predictors and covariates, no variance inflation factor was higher than 2, suggesting multicollinearity was not an issue. Models were fit in R [105] within the maximum likelihood framework using the lme function from the nlme package [106]. All test statistics were two-sided, and *P* values <.05 were considered statistically significant.

## Results

### Baseline Sample Characteristics

Sample characteristics are presented in Table 1. Hypertension/high cholesterol was the most common heart-related condition, and comorbid heart disease and diabetes was the least common. Users reported an average of 2 chronic conditions; other than the 3 heart-related conditions, the most commonly selected categories were insomnia, chronic pain, and “other.” Approximately three-quarters of users were female, and the most frequent age category was 45-54 years old. Mean total duration of time between first and last assessments was approximately 100 days, and the average number of activities completed per week between a user’s first and last assessments was 2.9 (SD 4.6), with a range of 0.04-29.94. In total, 636 users (636/1803, 35.27%) achieved the recommended use of 2 activities averaged per week.

**Table 1:** Baseline sample characteristics (N=1803).

Characteristics		Users
**Heart conditions, n (%)**		
	Hypertension/high cholesterol	1180 (65.44)
	Diabetes	361 (20.00)
	Hypertension/high cholesterol and diabetes	169 (9.37)
	Heart disease	69 (3.83)
	Heart disease and diabetes	24 (1.33)
**Gender, n (%)**		
	Female	1348 (74.76)
	Male	447 (24.79)
	Other	8 (0.44)
**Age group (years), n (%)**		
	18-24	61 (3.38)
	25-34	228 (12.65)
	35-44	394 (21.85)
	45-54	646 (35.83)
	55-64	474 (26.29)
User is in a relationship, n (%)		1340 (74.32)
User has at least one minor child, n (%)		569 (31.56)
Total chronic conditions, mean (SD)		2 (1)
**Total chronic conditions, n (%)**		
	Arthritis	222 (12.31)
	Asthma	201 (11.15)
	Cancer	40 (2.22)
	Chronic pain	419 (23.24)
	Eczema	106 (5.88)
	Insomnia	473 (26.23)
	Migraine	238 (13.20)
	Multiple sclerosis	15 (0.83)
	Postpartum depression	39 (2.16)
	Psoriasis	60 (3.33)
	Rheumatoid arthritis	50 (2.77)
	Other conditions	480 (26.62)
Total time between baseline and last assessment (days), mean (SD)		101 (43)
Total number of assessments, mean (SD)		4 (2)
Days between assessment, mean (SD)		50 (36)
Happify score at baseline, mean (SD)		46 (21)
GAD-2^a^ score at baseline, mean (SD)		3 (2)
Number of activities per week, mean (SD)		2.9 (4.6)
Recommended use^b^, n (%)		636 (35.27)

^a^GAD 2: Generalized Anxiety Disorder-2.

^b^Users met criteria for recommended use if they completed an average of 2 or more activities per week between their first and last assessments.

### Changes in Well-Being and Anxiety Over Time

For the Happify scale, the initial multivariable model without an interaction demonstrated an effect for time from baseline (*b=*0.049; 95% CI 0.041 to 0.057; *P*<.001), supporting our first hypothesis that users would report significant improvements in subjective well-being over time. Specifically, users were predicted to improve 0.049 points per day, amounting to about 2.1 points over 6 weeks or 8.8 points over 6 months.

**Figure 1:**
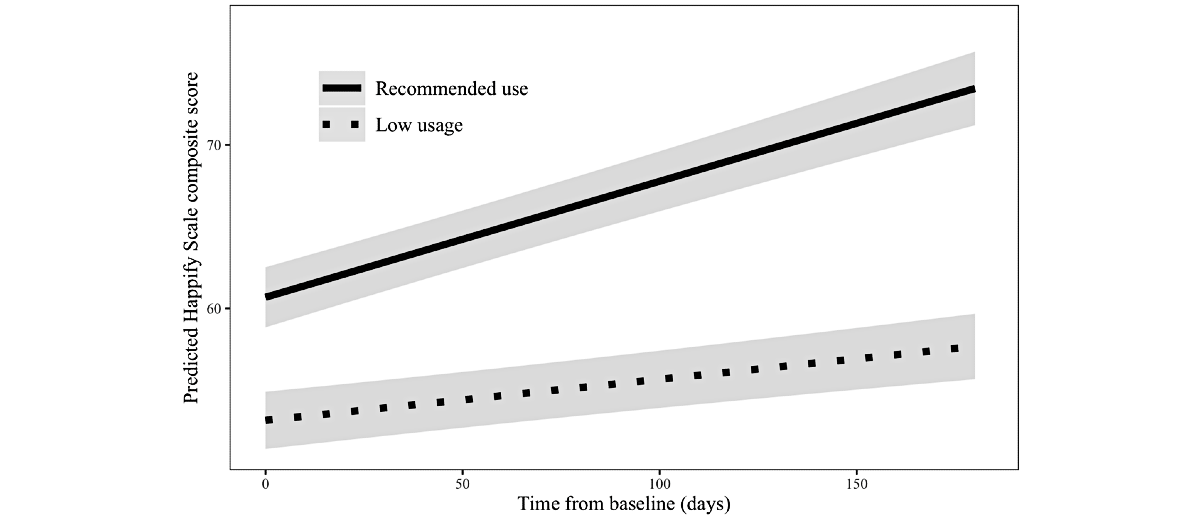
Change in predicted subjective well-being scores over time by recommended use.

There was also a main effect of whether a user completed the recommended number of activities: Those who used as recommended had higher Happify scale scores overall (*b*=9.071; 95% CI 7.563 to 10.578; *P*<.001). The interaction term between time and whether a user met the recommended use was significant as well (*b=*0.047; 95% CI 0.032 to 0.063; *P*<.001), supporting the second hypothesis that those who used Happify at or above the recommended use would experience significantly greater improvements in well-being over time than those who completed fewer activities. Individuals who averaged at least 2 activities per week would be expected to improve by 0.077 points per day (about 3.2 points over 6 weeks or 13.9 points over 6 months), whereas users who did not use as recommended would only be predicted to improve 0.028 points per day (about 1 point over 6 weeks or 5 points over 6 months). The interaction is depicted in Figure 1. Additionally, users with higher GAD-2 scores at baseline scored lower on the Happify scale overall, users with more chronic conditions scored lower, and users in a relationship scored higher. Table 2 presents estimates for both models.

For the GAD-2, there was also a significant main effect for time (*b=–*0.003; 95% CI –0.004 to –0.003; *P*<.001) for the initial model, supporting the first hypothesis that users would report significant improvements in anxiety over time. Specifically, users were predicted to improve by 0.003 points per day, amounting to 0.126 points over 6 weeks or 0.54 points over 6 months. There was also a main effect of whether a user completed the recommended number of activities: Those who met recommended use had lower GAD-2 scores overall (*b*=–0.362; 95% CI –0.484 to –0.239; *P*<.001).

**Figure 2:**
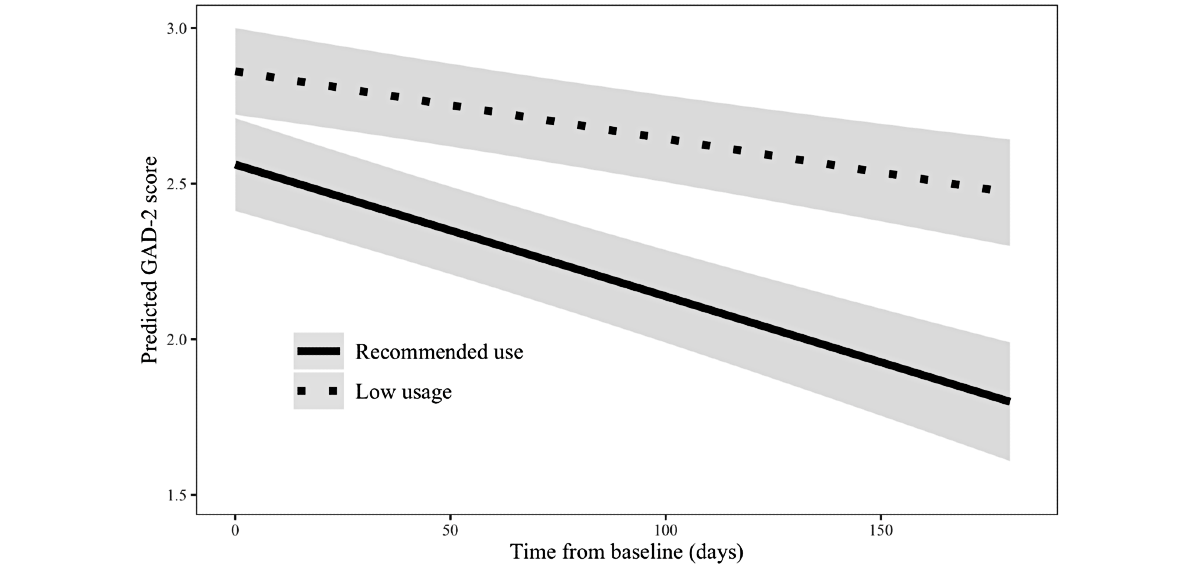
Change in predicted Generalized Anxiety Disorder-2 (GAD-2) scores over time by recommended use status.

The inclusion of the interaction term between time and recommended use was also significant (*b=*–0.002; 95% CI –0.004 to –0.001; *P*=.001), supporting the second hypothesis: Those who used Happify at or above the recommended use level experienced significantly greater reductions in anxiety over time compared with those who completed fewer activities. Individuals who averaged at least 2 activities per week would be expected to improve by 0.004 points per day (about 0.17 points over 6 weeks or 0.72 points over 6 months), whereas users who did not use as recommended would only be predicted to improve 0.002 points per day (about 0.084 points over 6 weeks or 0.36 points over 6 months). The interaction is depicted in Figure 2.

Additionally, female users scored higher (greater anxiety) on the GAD-2 overall, users with higher Happify Scale scores at baseline scored lower on the GAD-2, users with more chronic conditions scored higher, and users in a relationship scored lower. Finally, users aged 18-24 years, users aged 25-34 years, and users aged 35-44 years all reported higher overall anxiety scores than those aged 45-54 years. Users aged 55-64 years reported lower scores than those aged 45-54 years. Table 3 presents the estimates for both models.

**Table 2:** Linear mixed effect model of main effects and interaction between recommended use and time for Happify Scale scores.

Predictor		Main effects–only model		Interaction model	
		Model estimate (95% CI)	*P* value	Model estimate (95% CI)	*P* value
Intercept^a^		60.474 (57.721 to 63.226)	<.001	61.037 (58.277 to 63.796)	<.001
Recommended use		9.071 (7.563 to 10.578)	<.001	7.636 (6.053 to 9.219)	<.001
Time from baseline (days)		0.049 (0.041 to 0.057)	<.001	0.03 (0.021 to 0.04)	<.001
Total chronic conditions		–2.168 (–2.693 to –1.642)	<.001	–2.168 (–2.694 to –1.643)	<.001
GAD-2^b^ score at baseline		–5.267 (–5.657 to –4.877)	<.001	–5.26 (–5.65 to –4.871)	<.001
Female gender		0.676 (–1.001 to 2.354)	.43	0.691 (–0.987 to 2.368)	.42
**Age group (years)**					
	18-24	–0.512 (–4.714 to 3.69)	.81	–0.509 (–4.709 to 3.692)	.81
	25-34	–1.354 (–3.727 to 1.019)	.26	–1.366 (–3.739 to 1.007)	.26
	35-44	–0.759 (–2.739 to 1.221)	.45	–0.768 (–2.747 to 1.212)	.45
	45-54	-^c^	-^c^	-^c^	-^c^
	55-64	1.131 (–0.769 to 3.031)	.24	1.131 (–0.769 to 3.031)	.24
User is in a relationship		6.093 (4.39 to 7.797)	<.001	6.106 (4.403 to 7.809)	<.001
User has at least one minor child		–1.035 (–2.755 to 0.684)	.24	–1.024 (–2.743 to 0.695)	.24
Recommended use by time from baseline		N/A^d^	N/A	0.047 (0.032 to 0.063)	<.001

^a^Represents the score for a male user aged 45-54 years at baseline with 0 chronic conditions, no anxiety symptoms, not in a relationship, with no children, and who did not meet the recommended use of an average of 2 activities per week.

^b^GAD-2: Generalized Anxiety Disorder-2.

^c^Reference group.

^d^N/A: not applicable to the first model.

**Table 3:** Linear mixed effect model of main effects and interaction between recommended use and time for anxiety scores on the Generalized Anxiety Disorder-2 (GAD-2).

Predictor			Main effects–only model				Interaction model		
			Model estimate (95% CI)		*P* value		Model estimate (95% CI)		*P* value
Intercept^a^			4.165 (3.914 to 4.416)		<.001		4.075 (3.821 to 4.33)		<.001
Recommended use			–0.362 (–0.484 to –0.239)		<.001		–0.28 (–0.421 to –0.139)		<.001
Time from baseline (days)			–0.003 (–0.004 to –0.003)		<.001		–0.002 (–0.003 to –0.001)		<.001
Total chronic conditions			0.135 (0.092 to 0.178)		<.001		0.137 (0.094 to 0.18)		<.001
Happify score at baseline			–0.041 (–0.043 to –0.038)		<.001		–0.039 (–0.042 to –0.036)		<.001
Female gender			0.161 (0.026 to 0.297)		.02		0.165 (0.029 to 0.301)		.02
**Age group (years)**									
	18-24	0.615 (0.274 to 0.955)		<.001		0.622 (0.278 to 0.965)		<.001	
	25-34	0.378 (0.186 to 0.569)		<.001		0.383 (0.191 to 0.576)		< .001	
	35-44	0.246 (0.086 to 0.405)		.003		0.241 (0.08 to 0.401)		.003	
	45-54	-^b^		-^b^		-^b^		-^b^	
	55-64	–0.163 (–0.316 to –0.01)		.04		–0.159 (–0.313 to –0.005)		.04	
User is in a relationship			0.181 (0.043 to 0.32)		.01		0.164 (0.025 to 0.304)		.02
User has at least one minor child			–0.007 (–0.146 to 0.132)		.93		0.003 (–0.137 to 0.143)		.96
Recommended use by time from baseline			N/A^c^		N/A		–0.002 (–0.004 to –0.001)		.001

^a^Represents the score for a male user aged 45-54 years at baseline with 0 chronic conditions, a score of 0 on the Happify Scale, not in a relationship, with no children, and who did not meet the recommended use of an average of 2 activities per week.

^b^Reference group.

^c^N/A: not applicable to the first model.

## Discussion

### Principal Findings

The objective of this analysis of real-world data from Happify users with self-reported CVD risk factors, including high blood pressure and cholesterol, diabetes, and heart disease, was to examine whether (H1) these users would report improvements in subjective well-being and anxiety over time and (H2) use of Happify as recommended would be associated with significantly greater improvement in subjective well-being and anxiety over time compared with less-than-recommended usage. Both hypotheses were supported. As predicted, users experienced significant improvement in subjective well-being and anxiety over time. However, in single-arm studies, such improvements could be attributed to factors other than the intervention, including regression to the mean, spontaneous remission, changes in a person’s medication or treatment regimen, or any number of other factors. We also found a significant interaction between recommended use (ie, completing at least 2 activities per week or not) and time for both subjective well-being and anxiety, which provides preliminary evidence that the changes in mental health outcomes may be due, at least in part, to the engagement with the intervention itself (though it is not, of course, dispositive). As shown in Figures 1 and 2, the *rate of improvement* was greater (faster) for those who met or exceeded the recommended use criteria compared with those who did not. This suggests that the observed improvements in mental health outcomes were not due to the passage of time alone, thereby reducing the influence of one potential confound and increasing the likelihood that the observed changes were due at least in part to the intervention.

### Strengths and Limitations

This study provided important extensions to prior research and included many strengths, but also several limitations. First, as this was a study of real-world data from Happify users, there was no opportunity to assign participants to a control group, and thus, we cannot determine whether the observed changes in outcomes were simply due to the passage of time, chance, or any number of other confounding factors. However, though RCTs are considered the gold standard for assessing the efficacy and safety of therapeutic interventions, naturalistic studies provide important insight into the performance of these interventions in real-world settings [107]. Real-world studies are free from many of the constraints imposed by more controlled research, such as strict eligibility criteria, which can limit the diversity of participants and the generalizability of the results [108]. As such, real-world studies have been recognized for their value by regulatory bodies such as the Food and Drug Administration [109]. This sample consisted of existing Happify users and so was not limited to those who could be reached via traditional research recruitment channels, nor was compensation given to users for participating in the study or using the app, removing the potential for such incentives to influence users’ behaviors. Therefore, this naturalistic analysis yields results that are more readily generalizable to the broader population of users than would be the case for a more controlled trial.

Second, despite these advantages regarding generalizability, the users retained for analysis may not be representative of Happify users as a whole. Although these data provide important insights into real-world use, users could easily stop using the app with little friction, resulting in a high dropout rate. This means that only a small proportion of potentially eligible users was included in the analysis, and they may systematically differ from other Happify users in their behaviors or dispositions. Unfortunately, this is a widespread issue, as the majority of open-access, digital mental health interventions are plagued by low usage and retention rates, with the median app losing 97% of its users within 30 days [110]. Studies of such interventions also suffer from high attrition and dropout, especially for observational or real-world evidence studies [111]. 

Another challenge to the generalizability of the study is regarding the gender balance within the sample. Approximately three-quarters of the sample were women, a trend that is common across many studies of digital mental health interventions [112] and consistent with prior research of Happify [90]. This may reflect the fact that proportionally more women are affected by a number of mental health issues than men [113] and/or that women may be more willing to engage with digital mental health interventions [114,115]. However, men, on average, are at higher absolute risk of developing CVD than women (although the relative risk of CVD morbidity and mortality is higher in women than men) [116]. Therefore, our study undersampled men relative to the proportion of men who experience CVD risk in the general population, and future studies should seek more representative samples. At the same time, the sample is likely biased toward those who are naturally more inclined to use digital mental health interventions, and consequently, our sample may be more closely representative of the people with CVD risk factors who would be most likely to actually use digital mental health interventions.

Despite these difficulties, this study demonstrated fairly high rates of usage relative to other studied interventions. After excluding those who merely downloaded the app but did not use it, which was only 3.51% (664/18,905) of the initial sample of users with CVD risk factors, we found that the average number of activities per week completed between a user’s first and last assessments was 2.9 and that 35.27% (636/1803) of the sample met criteria for recommended use. This is actually quite high, especially considering that in this and prior research, a significant improvement has been observed in those who complete as few as 2 activities per week over 6-8 weeks [90,91]. Most Happify activities take between 2 minutes and 15 minutes to complete. Given the many demands on people’s time, in particular those with poor cardiovascular health, who may experience higher stress levels [117] and impaired physical function [118], digital mental health interventions that are effective with minimal usage and time commitment are especially valuable.

Third, we were limited to the use of only 2 brief, self-reported measures of mental health capturing subjective well-being and anxiety that were part of the general user experience of the existing Happify product. These 2 measures alone are clearly not representative of all features of mental health, but we were unable to add other measures due to the naturalistic nature of the study. Although the Happify Scale has been observed to be highly correlated *(r*=–0.70) with validated measures of depression [97], it is not a direct measure of depression or other important mental health factors, like stress. Additionally, the GAD-2 is less responsive to change than a longer, more sensitive measure of anxiety like the GAD-7 [119] and therefore more precise and potentially larger effects for anxiety might have been observed had we used a more psychometrically robust measure. Finally, all other data was self-reported, and thus, we cannot confirm whether users’ conditions were official diagnoses.

### Future Directions

Additional research is needed to address these limitations and more fully understand how Happify and other digital mental health interventions can improve mental and physical health outcomes in patients with CVD risks. Existing research suggests that interventions designed to improve mental health may also lead to reduced CVD risk [64], but to determine whether an intervention like Happify has a direct causal impact on cardiovascular health, an RCT would be necessary. Additionally, the version of Happify studied here was not specially tailored or personalized to the particular population of users with CVD risks. Tailored interventions have been shown to improve outcomes over and above nontailored ones [120], and thus, there is an opportunity to create and test an intervention that addresses the unique challenges and concerns of users with CVD risk factors. Furthermore, there is great potential in the integration of sensors and wearable technology into interventions for CVD risk monitoring and prevention, which allow for greater insight into health behaviors and opportunity to impact users in real time [121]. Other technology, such as artificial intelligence, could be used to process this large influx of data and provide ongoing recommendations that are tailored to meet individual user’s needs without dramatically increasing the burden of care [75]. These and other technologies should be further explored to determine what kinds of interventions best serve this population. Future RCTs and other studies should also expand the measures of mental health beyond those studied here to include validated measures of quality of life, depression, and stress, all of which have been associated with CVD risk [122], and there is a further opportunity to address some of the weaknesses associated with self-reported measures by incorporating data from wearables or other devices like home blood pressure monitors on physiological outcomes [19]. Finally, future research should evaluate the impact of digital mental health interventions on mental health and CVD risk factors beyond the current window of 6 months to establish the long-term trajectory and durability of the effects.

### Conclusions

This retrospective analysis of real-world data provides preliminary evidence that Happify users with self-reported CVD risk factors including high blood pressure or cholesterol, diabetes, and heart disease experienced improved well-being and anxiety over time and that those who used Happify at or above the recommended level experienced greater improvements over time in these aspects of mental health than those who completed fewer activities. This study assessed data from a publicly available digital mental health product, and consequently, the regularity of data collection and consistency of usage were highly variable. Although greater Happify usage was significantly associated with greater improvements in mental health (thereby reducing the likelihood that observed changes were due to maturation effects), the lack of a control group nevertheless makes it impossible to draw direct causal links between Happify usage and improvements in mental health. Acknowledging these limitations, our findings extend previous research, which demonstrated that engagement with Happify as recommended was associated with improved well-being among physically healthy users [90,123] and in those with chronic conditions [91], to a new population for whom mental health is especially critical: those at risk of developing CVD. Also, by including both subjective well-being and anxiety symptoms as outcomes, this study provides insight into a broader understanding of mental health than assessed in previous naturalistic studies of Happify [91]. Understanding how to increase positive experiences and reduce negative ones is essential to achieving the flourishing of the “whole person” [124], and digital mental health interventions appear to be a promising means of supporting people, including those with CVD risk factors, in this pursuit [125].

## Abbreviations

CBT: cognitive behavioral therapy
CVD: cardiovascular disease
GAD-2: Generalized Anxiety Disorder-2
LME: linear mixed effects
RCT: randomized controlled trial
